# Tackling the Research Challenges of Health and Climate Change

**DOI:** 10.1289/ehp.0901171

**Published:** 2009-12

**Authors:** Roger Glass, Joshua Rosenthal, Christine M. Jessup, Linda Birnbaum, Chris Portier

**Affiliations:** Fogarty International Center, National Institutes of Health, Department of Health and Human Services, Bethesda, Maryland, E-mail: joshua_rosenthal@nih.gov; National Institute of Environmental Health Sciences, National Institutes of Health, Department of Health and Human Services, Research Triangle Park, North Carolina

Ebi et al. (2009) presented a timely and important analysis of the federal investment in research focused on understanding, avoiding, preparing for, and adapting to the health impacts of climate variability and change. The authors argued that the public health community is inadequately prepared to address the health risks associated with climate variability and change, and that funding necessary to address this challenge is inadequate. Ebi et al. (2009) were particularly critical of the National Institutes of Health (NIH) for overstating its investments in research on the health impacts of climate change, citing a 2007 NIH spending report of $164 million for Health Effects of Climate Change. We would like to respond by highlighting two current activities of the NIH that address these issues: the Trans-NIH Working Group on Climate Change and Health (led by the FIC) and an interagency working group on climate change and health (led by the National Institute of Environmental Health Sciences). Both activities are in midstream, but we plan to have initial products and recommendations available by the fall 2009.

In 2008, a planning group was convened at the NIH to assess the research questions in health and medicine that climate change presents. Sixteen NIH institutes and centers are actively participating in the Trans-NIH Working Group on Climate Change and Health, with coordination from the Fogarty International Center (FIC). The working group is *a*) analyzing the relevance of the NIH portfolio in this area; *b*) engaging the biomedical research community in a discussion of the health effects of climate change; and *c*) identifying research needs and priorities for an NIH research agenda for climate change and health, including the development and evaluation of clinical and public health strategies for adaptation to a changing world.

In January 2009, an interagency working group was formed to identify areas in which strategic research on the linkage between climate change, the environment, and human health could greatly enhance our understanding. Led by the NIEHS, this group was formed to expand the activities of the NIH-focused activity and aid in the coordination of a broader research effort focused on human health for the entire U.S. government research community. The working group is *a*) examining the research portfolio on the health impacts of climate change across the U.S. government; *b*) expanding the dialogue among federal agencies to help coordinate the diverse missions of the U.S. government agencies; and *c*) developing a general conceptual model for research needs to aid in research coordination. The results of this interagency working group, when combined with the Trans-NIH Working Group, will guide the NIH in developing a research portfolio that is science driven and directly relevant to the needs for prevention and intervention to protect human health from climate change.

Assessing the relationship of basic research projects to policy-defined problems is often challenging. For climate change and biomedical research, the challenge is compounded by the complexity of the interaction pathways between climate variables, environmental change, and human health outcomes. Furthermore, concerns over the nature and magnitude of the health threats have changed considerably in the past few years. The figures cited by the NIH for Health Effects of Climate Change in recent years reflected studies that are principally basic human biology related to conditions that are sensitive to climate and atmospheric phenomena, including ultraviolet radiation, To provide an analysis of the NIH portfolio that is more relevant to the current policy concerns with effects of global warming, we are utilizing the new NIH grant fingerprinting technology [Research, Condition, and Disease Categorization (RCDC)] to capture all the potentially relevant projects, followed by a manual process in which experts from the institutes and centers that administer the grants categorize this diverse pool of projects into three general bins: *a*) those with a climate change focus, *b*) those that address climate parameters, and *c*) those that address human conditions that are climate sensitive. Details on the methods and results are forthcoming, but preliminary results indicate that only a handful of research projects in the 2008 portfolio (< 10) had a direct focus on the health effects of interannual or long-term climate change, a somewhat larger group (90–100) studied health effects in relation to climate parameters, and the largest group (> 700) were indirectly climate-relevant in that they focused on the basic human biology of climate-sensitive conditions without actually examining climate parameters. This detailed project analysis essentially agrees with the view of Ebi et al. (2009) that there are relatively few research projects directly focused on the interface of health and climate change, and thus the investment by the agency, using this narrower definition, is significantly less than the $164 million reported by NIH in 2007 for “Health Effects of Climate Change” (NIH 2009a).

In parallel with this portfolio analysis, we have begun to assess—through both the Trans-NIH Working Group and the inter-agency working group—what the NIH research agenda for climate and health should look like. These efforts will unfold over the next months, but in general terms, we expect the recommendations to include the need to *a*) understand the etiology and epidemiology of current and future health threats from global climate change; *b*) identify the most vulnerable populations/subpopulations and their specific health and medical concerns; *c*) develop predictive models with enough resolution to inform surveillance and medical and public health planning; *d*) develop clinical, translational, and implementation science tools, including cost-effectiveness estimates, to prevent and/or intervene on principal health concerns; and *e*) enhance the human research capacity necessary to advance these goals.

Importantly, the NIH has already taken steps to address two key needs identified in our preliminary analysis—predictive modeling of potential health effects of climate change, and capacity building in environmental public health—through soliciting grants in this area as part of the Challenge Grants initiative enabled by the American Recovery and Reinvestment Act (ARRA 2009; NIH 2009b). To facilitate public health planning and inform adaptation strategies, we need to develop quantitative and predictive models of effects of climate change and of the burden related to a diversity of communicable and non-communicable diseases, as well as enhanced research capacity through skills and partnerships with communities.

The points raised by Ebi et al. (2009) are important and appreciated by the NIH community. Although the overall climate- relevant health research portfolio of the agency has been significant, there has been very little NIH-supported research directly focused on health effects of global climate change. The NIH has the scientific and administrative capability to address the scientific issues and the fundamental responsibility for supporting biomedical and public health research at U.S. academic centers where most of the relevant research will be done. Given the enormity and complexity of this issue and the important role of the NIH in health research, both in the United States and around the world, it is essential that the NIH be more actively focused on the health implications of climate change and the science that will help us adapt to these challenges.
